# The analysis of percutaneous pedicle screw technique with guide wire-less in lateral decubitus position following extreme lateral interbody fusion

**DOI:** 10.1186/s13018-019-1354-z

**Published:** 2019-09-05

**Authors:** Akihiko Hiyama, Daisuke Sakai, Masato Sato, Masahiko Watanabe

**Affiliations:** Department of Orthopaedic Surgery, Surgical Science, Tokai University School of Medicine, 143 Shimokasuya, Isehara, Kanagawa 259-1193 Japan

## Abstract

**Background:**

Lateral lumbar interbody fusion (LLIF) and bilateral percutaneous pedicle fixation are valuable, minimally invasive lateral approaches used to treat symptomatic degenerative disc disease. In the current procedure, the patient’s position on the operating table is changed after LLIF surgery from the lateral decubitus to the prone position. The ability to perform both approaches with the patient in the same position should reduce operation time. Use of a guide wire is problematic during percutaneous pedicle screw (PPS) insertion using fluoroscopy with the patient in the lateral decubitus position. A new guide wire-less PPS system may solve this problem and reduce operation time. Here, we evaluated the operative data and efficacy for this technique.

**Methods:**

This study included 30 patients (aged 70.8 ± 8.5 years; 17 men, 13 women) who underwent a combined operation (indirect decompression) using extreme lateral interbody fusion (XLIF) with only a single level for lumbar spinal canal stenosis and lumbar degenerative spondylolisthesis. Patient demographics and operative data were compared between two groups: patients who remained in the lateral decubitus position for pedicle screw fixation (L group) and those turned to the prone position (P group). Radiographic assessment was performed using pre- and postoperative anteroposterior and lateral lumbar films with measurement of lumbar lordosis, segmental lordosis, and segmental translation.

**Results:**

We analyzed 18 patients in the P group and 12 in the L group. Age, sex, height, body weight, body mass index, estimated blood loss, and length of stay did not differ between groups. The operation time was 34 min shorter for the L group (P group 111.9 ± 25.0 vs. L group 77.5 ± 22.2 min, *p* < 0.01). Pre- and postoperative lordosis, segmental lordosis, and segmental translation did not differ significantly between groups.

**Conclusions:**

A single position after XLIF surgery is a feasible modification to the standard procedure when used with fluoroscopy and a guide wire-less PPS system. The time saved is the main advantage of inserting the PPS with the patient in the lateral decubitus position without repositioning. Use of the lateral PPS with a guide wire-less technique may help improve operative efficiency and reduce cost.

## Background

Of the minimally invasive lateral lumbar interbody fusion (LLIF), extreme lateral interbody fusion (XLIF) and oblique lumbar interbody fusion (OLIF) are techniques used for indirect decompression of the neural structures through interbody distraction and fusion in the lumbar spine. Good results have been reported for these techniques [1–4].

Concomitant posterior fixation is often recommended because of the higher rates of nonunion after the LLIF procedure. In this case, the use of bilateral percutaneous pedicle screws (PPSs) is considered to be the gold standard [5]. In the current procedure, after the lateral access surgery, the patient is repositioned in the prone position for the pedicle screw fixation. This repositioning requires completing a second round of preparation, draping, and room positioning, which increases the operation time and cost because of the extra use of materials.

Some groups have described a technique in which the PPSs are placed while the patient remains in the lateral position following the LLIF. Ziino et al. reported that lateral PPS fixation following LLIF decreases the operating room time without compromising postoperative lordosis or the complication rate [6]. Lateral PPS surgery using three-dimensional (3D) neuronavigation, such as the O-arm system and navigation, has been reported [7]. However, few institutions have installed these instruments because of the cost.

**Fig. 1:**
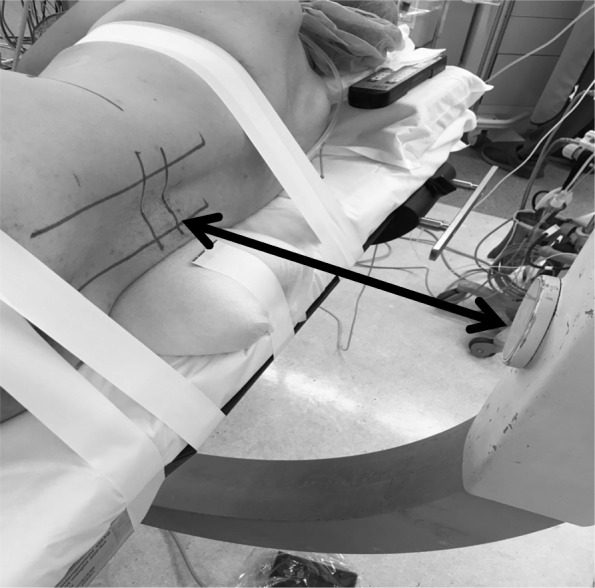
Lateral decubitus position. During PPS insertion with a guide wire, it is difficult to secure a working space (arrow) between the patient and the fluoroscopy, and the use of fluoroscopy during insertion of PPS with the patient in the lateral decubitus position can be awkward for the surgeon

The current procedure is to insert the PPS through the guide wire, and the setup is cumbersome in most cases. That is, it can be difficult to secure a working space between the patient and the fluoroscopy, and use of fluoroscopy during insertion of PPSs with the patient in the lateral decubitus position can be awkward for the surgeon (Fig. 1). With this as background, we thought that a lateral repositioning procedure (XLIF + lateral PPS) using a guide wire-less PPS system (Viper Prime^TM^, DePuy Synthes Spine, Raynham, MA, USA) may be useful for solving these problems. In this study, we compared the operation time, amount of bleeding, and complications between patients who were in the prone position during PPS insertion (P group) with those who received PPS insertion after the conventional position change to the lateral decubitus position (L group). We also describe the lateral PPS procedure using guide wire-less PPS.

## Materials and methods

### Included patients

The inclusion criteria were patients aged > 18 years who were undergoing a combined operation (indirect decompression) using XLIF with only single-level lumbar spinal canal stenosis (LCS) and lumbar degenerative spondylolisthesis (DS) at a single institute from January 2016 to June 2019. We conducted our retrospective review using a retrospective cohort. The exclusion criteria included patients who had undergone previous lumbar spinal surgery or those who were undergoing combined procedures including direct posterior decompression and posterior lumbar fusion.

In total, 30 patients (aged 70.8 ± 8.5 years; 17 men, 13 women) were included. The patient demographics (age, sex, height, body weight, and body mass index (BMI)) and operative data (blood loss, operation time, and change in hemoglobin (Hb) level from before to the first day after surgery) were recorded. Radiographic assessment was performed using pre- and postoperative anteroposterior (AP) and lateral lumbar films; the lumbar lordosis, segmental lordosis, length of stay, and intraoperative complication rate were recorded.

### Operative technique (XLIF and PPS fixation)

Interbody fusion was completed using the XLIF technique as described by Ozgur et al. [3]. Briefly, the patient was placed in the lateral decubitus position with the hip at the level of the break in the operating table. The chest and hip areas were secured to the table with tape. A previous report has suggested that the patient’s position is critical, and there was a slight learning curve for surgeons when performing the surgery involving insertion of the “downside” PPS with the patient in the lateral decubitus position [8]. For example, positioning the patient too far from the edge of the bed can limit the surgeon’s ability to place the hand low enough to medialize the downside pedicle screws. However, this is less of a concern when performing a lateral PPS procedure using the Viper Prime^TM^.

Once the position was decided, the XLIF was performed according to the previous method [3]. This facilitates access to the largest number of disc spaces with a relatively small incision. Blunt dissection was then used to access the disc spaces under fluoroscopic guidance. After removal of the disc material with a rongeur, a Cobb elevator was advanced gently under fluoroscopy guidance along the endplates to release the contralateral annulus. Cage size trials were followed by additional disc curettage and rasping of the endplates. All cages were inserted using two containment sliders to protect the endplates and to keep the graft material inside the cage. For all patients, the side-to-side cage size was decided according to the width of the endplates at that level based on intraoperative fluoroscopic guidance, and titanium cages with a standard 18 mm width were used. The maximum distraction achieved during discectomy using the trial inserts provided guidance as to the height of the cage. The choice of these XLIF cages (CoRoent XL; NuVasive Inc., San Diego, CA, USA) was decided by the surgeon. Cage lengths ranged from 45 to 55 mm, and heights from 8 to 12 mm.

**Fig. 2:**
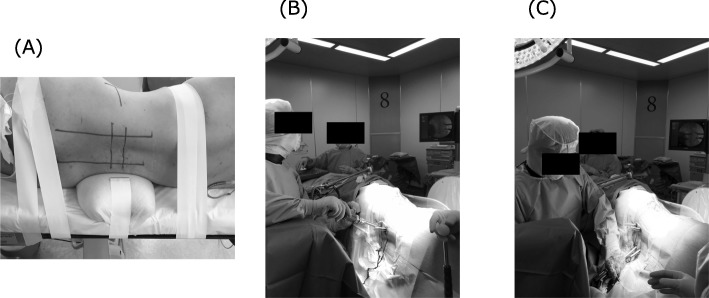
**a** Intraoperative images demonstrating placement of the PPS using the Viper Prime^TM^ with the patient in the lateral decubitus position. Marking the location of the incision. **b** Upside PPS placement. **c** Downside PPS placement

The lateral PPS technique using the Viper Prime^TM^ instrument is shown in Fig. 2. Following the XLIF, patients in the P group were turned to the prone position and then prepared again and draped. Bilateral PPS surgery was then performed with the patient in the prone position. Patients in the L group remained in the lateral decubitus position for PPS fixation. An image in the AP view was taken to mark the lateral radiographic borders of the pedicles for screw placement. Using a lateral view, the center of each pedicle was identified and marked. A small incision 2–3 cm lateral to the lateral radiographic borders of each pedicle was made for percutaneous exposure, and the stylets were then docked at the junction of the transverse process and the superior articular process. The stylets were then inserted with a hammer to hold the spot within the pedicles. After the stylets were inserted into the pedicle inner rim, an image in the AP view was taken to confirm in the lateral view that the posterior body wall had been reached. At that point, the C-arm was then brought to a lateral position to maximize the working space for screw placement. After all screws had been inserted, a rod was passed percutaneously and secured to the screw heads using setscrews.

### Statistical analysis

Statistical analyses were performed using IBM SPSS Statistics version 20.0 (IBM Corp., Armonk, NY, USA). All values are expressed as mean ± standard deviation. Univariate differences between the L and P group were assessed using independent-sample *t* tests or the Mann–Whitney *U* test for data that were not normally distributed. For all statistical analyses, the type 1 error was set at 5% and *p* < 0.05 was considered to be significant.

## Results

All patient characteristics and operative details are given in Table 1. The indications for lumbar interbody fusion surgery in this study included degenerative spondylolisthesis (*n* = 14) and lumbar canal stenosis (*n* = 16). Four patients underwent fusion at L2–3, 8 patients at L3–4, and 18 patients at L4–5. The operation times ranged from 54 to 152 min (mean, 98.2 ± 29.1 min). Operative blood loss ranged from 4 to 332 ml (mean, 54.4 ± 70.1 ml). The length of hospitalization ranged from 9 to 26 days (mean, 16.3 ± 4.5 days). There were no intraoperative complications related to general condition, and no patients underwent reoperation for mispositioned pedicle screws. Intraoperative endplate injury was identified in 5 of the 30 patients (16.7%). Age, sex, height, body weight, BMI, Hb level, estimated blood loss, and length of stay did not differ between the L and P groups. However, the operation time was 34 min longer for the P group (111.9 ± 25.0 min) than for the L group (77.5 ± 22.2 min, *p* < 0.01) (Table 2). No significant differences were observed between the pre- and postoperative lumbar lordosis, segmental lordosis, change in lumbar lordosis or segmental lordosis, or segmental translation (Table 3).

**Table 1:** Demographic and clinical data

Patients (*n*)	30
Age (years)	70.8 ± 8.5
Female	13 (43.3%)
Height (cm)	159.9 ± 9.3
Body weight (kg)	62.4 ± 11.7
Body mass index (kg/m^2)^	24.5 ± 3.5
Diagnosis	
LCS	16
DS	14
Spine levels	
L1–2	0 (0%)
L2–3	4 (13.3%)
L3–4	8 (26.7%)
L4–5	18 (60.0%)
Blood loss (ml)	54.4 ± 70.1
Time in operating room (min)	98.2 ± 29.1
Length of stay (days)	16.3 + 4.5

*LCS* lumbar spinal canal stenosis, *DS* lumbar degenerative spondylolisthesis. All values are in mean ± standard deviation

**Table 2:** Comparison of two groups

Characteristic	P group (*n* = 18)	L group (*n* = 12)	*p* value
Age (years)	69.7 ± 7.3	72.4 ± 10.2	0.305
Sex (M, F)	11, 7	6, 6	0.632
Height (cm)	160.8 ± 9.3	158.7 ± 9.5	0.662
Body weight (kg)	63.5 ± 13.1	60.9 ± 9.7	0.415
Body mass index (kg/m^2^)	24.6 ± 3.6	24.2 ± 3.5	0.787
Blood loss (ml)	69.0 ± 83.8	32.4 ± 35.0	0.095
Pre-ope Hb (g/dl)	13.6 ± 1.9	14.2 ± 1.1	0.439
First post-ope Hb (g/dl)	12.1 ± 2.1	12.6 ± 1.5	0.545
Change in Hb pre-ope to first post-ope (g/dl)	− 1.6 ± 1.0	− 1.6 ± 0.9	0.983
Time in operating room (min)	111.9 + 25.0	77.5 + 22.2	< 0.01
Length of stay (days)	16.0 + 4.2	16.8 + 5.1	0.573

All values are in mean ± standard deviation

*pre-ope* preoperative, *post-ope* postoperative

**Table 3:** Radiological outcomes between two groups

Characteristic	P group (*n* = 18)	L group (*n* = 12)	*p* value
Pre-ope lumbar lordosis (degree)	33.9 ± 12.8	30.7 ± 11.3	0.545
Post-ope lumbar lordosis (degree)	34.8 ± 11.7	28.3 ± 10.3	0.158
Change in lumbar lordosis pre-ope to post-ope (degree)	1.7 ± 7.5	− 2.4 ± 7.1	0.158
Pre-ope segmental lordosis (degree)	3.0 ± 4.6	4.0 ± 5.6	0.285
Post-ope segmental lordosis (degree)	5.7 ± 4.3	6.4 ± 3.6	0.465
Change in segmental lordosis pre-ope to post-ope (degree)	2.3 ± 4.5	2.4 ± 3.0	0.851
Pre-ope segemental translation (mm)	2.0 ± 5.4	4.1 ± 4.0	0.200
Post-ope segmental translation (mm)	1.2 ± 3.9	1.5 ± 2.5	0.755
Change in segmental translation pre-ope to post- ope (mm)	0.8 ± 2.8	2.5 ± 2.1	0.072

All values are in mean ± standard deviation

*pre-ope* preoperative, *post-ope* postoperative

## Discussion

Since the introduction of the PPS technique in Japan in 2005, posterior spinal surgery has become less invasive, and PPSs are now used widely in the surgical treatment of infections, tumors, and trauma [9–11]. The new Viper Prime^TM^ is characterized by integration of the screw and stylet. Until now, it has been difficult to access the downside PPS insertion angle with the patient in the lateral decubitus position because of the limited working space between the operating table and fluoroscopy. The new PPS system allows the surgeon to create a working space between the operating table (patient’s back) and the fluoroscopy which has not been possible in earlier third-generation PPS systems that require insertion of a hollow PPS through the guide wire. Using the Viper Prime^TM^ with the patient in the lateral decubitus position allows the PPS to be inserted easily.

Previous studies have shown that pedicle screw insertion using 3D neuronavigation is more secure than conventional insertion techniques [12–14]. Instruments such as computed tomography (CT) fluoroscopy (O-arm system) and navigation, which image the anatomical structures of the spine during surgery, are not available at many facilities. In the lateral decubitus position, the Viper Prime^TM^ system can be used to insert PPSs with the patient in the lateral decubitus position because the fluoroscope can be used without the need for additional specialized medical instruments.

**Fig. 3:**
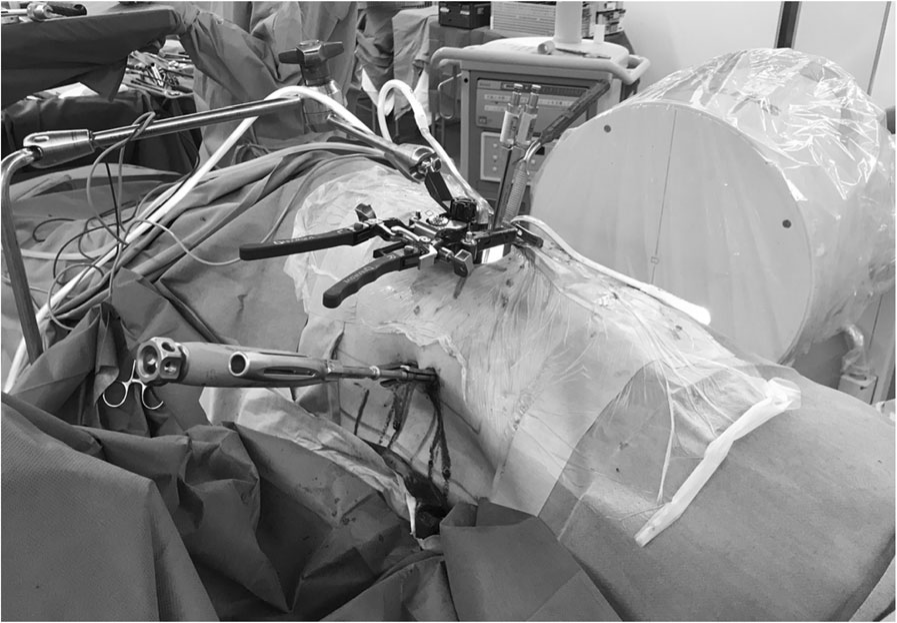
Correction of lumbar spondylolisthesis using XLIF and upside PPS with the patient in the lateral decubitus position

One advantage of the guide wire-less PPS system is that because the PPS can be inserted with the patient in the lateral decubitus position without the need for repositioning, this method may be used to correct vertebral slippage at the same time as the XLIF. The trick in compensation for slippage is to insert the upside PPS first, pass the rod a little further than the measurement, and then use the XLIF approach to remove the disc material. This is needed because the special operative window needed for XLIF interferes with fluoroscopy, and it can be difficult to evaluate the position of the screw and rod by fluoroscopy. After the disc is removed, the slip is corrected, the caudal setscrew is loosened, the XLIF cage is then inserted, and the downside PPS is inserted. In the future, we plan to determine how much correction can be made with this technique (Fig. 3).

The second advantage is shortening of the operation time, which should also improve the patient’s experience by hastening their return home and recovery. In the current study, hospital stay was an average of 16 days. Japanese social healthcare system may have influenced the length of hospital stays. As a reason for this, although not all, we basically aim at about 2 weeks as an indication of hospital stay including a rehabilitation period when elderly patients can return home. In addition, it is thought that the hospital stay might have been extended because dialysis patients and diabetes mellitus patients were included. On the other hand, the average total mean operation time was 77 min and blood loss was 32 ml with the guide wire-less method. The difference in operation time was 34 min between the prone PPS and the lateral PPS. Our results are consistent with those reported by Tohmeh et al. [15], who found an average difference of 38 min (range 30–84 min) in the repositioning time between the lateral decubitus and prone positions. In a study of the use of lateral PPSs by Blizzard and Thomas [8], 72 patients were treated with lateral interbody fusion and PPS in the lateral decubitus position. A mean total operation time of 87.9 min (1.1 fused levels per patient) and blood loss of 53 ml were found. They reported a shorter operation time with PPS insertion in the lateral decubitus position but a similar PPS breach rate as that reported previously in the prone position [16–18]. In our study, we did not analyze the PPS breach rate, but none of the patients experienced paralysis or leg pain after surgery or required reoperation to replace the screws. The third advantage of the guide wire-less method is that there is no risk of guide wire problems [19] given that the Viper Prime^TM^ system does not use guide wires. Another advantage of the PPS insertion method with the patient in the lateral decubitus position method is that patient repositioning is not required, which should reduce medical costs [8]. Although calculating the cost of each minute in the operating room is complex, the use of the lateral PPS technique after the LLIF may reduce cost by reducing the time in the operating room.

The limitations of this study are the retrospective design and that all lateral PPS surgeries were performed at a single center by a single surgeon. Although the latter may have introduced some bias to our study, we feel that inclusion only of patients treated by a single surgeon created a more uniform cohort for study, especially given that the data were collected from a series of cases. Another limitation is that the sample size was very small. However, to our knowledge, this is the first report on the use of the lateral PPS and fluoroscopy with a guide wire-less technique without patient repositioning after XLIF surgery. Despite the small sample size, we found that this technique significantly shortened the operation time. In addition, we did not evaluate long-term clinical outcomes and fusion rates. We think that further studies are needed to evaluate the effectiveness as well as the short- and long-term impact of this method on clinical outcomes and fusion rates compared to conventional surgical approaches.

A final limitation of this study is the lack of information about the insertion accuracy for the lateral PPSs. CT scans were not routinely obtained postoperatively to assess the fusion or screw position because no patients exhibited postoperative complications related to insertion of the screws, and we could not justify the risk of exposure to further CT imaging.

## Conclusion

In this study, the all-lateral technique requiring a single patient position after XLIF surgery is a feasible modification of the standard procedure used with fluoroscopy and new PPS systems. Operative blood loss, length of hospital stay, and correction of lordosis did not differ between patients treated with the all-lateral technique and those who were repositioned in the standard procedure. However, the operation time was reduced by 34 min in the all-lateral technique. This new technique reduces the time and need for staffing associated with intraoperative repositioning and may lead to significant cost savings.

## Data Availability

Data are available upon request from the corresponding author.

## Abbreviations

3D: Three-dimensional
AP: Anteroposterior
BMI: Body mass index
Hb: Hemoglobin
LLIF: Lateral lumbar interbody fusion
OLIF: Oblique lumbar interbody fusion
PPS: Percutaneous pedicle screw
